# Making and monitoring errors based on altered auditory feedback

**DOI:** 10.3389/fpsyg.2014.00914

**Published:** 2014-08-20

**Authors:** Peter Q. Pfordresher, Robertson T. E. Beasley

**Affiliations:** Auditory Perception and Action Laboratory, Department of Psychology, University at Buffalo-State University of New YorkBuffalo, NY, USA

**Keywords:** auditory feedback, error monitoring, sequence production, music performance, poor-pitch singing, internal models

## Abstract

Previous research has demonstrated that altered auditory feedback (AAF) disrupts music performance and causes disruptions in both action planning and the perception of feedback events. It has been proposed that this disruption occurs because of interference within a shared representation for perception and action (Pfordresher, 2006). Studies reported here address this claim from the standpoint of error monitoring. In Experiment 1 participants performed short melodies on a keyboard while hearing no auditory feedback, normal auditory feedback, or alterations to feedback pitch on some subset of events. Participants overestimated error frequency when AAF was present but not for normal feedback. Experiment 2 introduced a concurrent load task to determine whether error monitoring requires executive resources. Although the concurrent task enhanced the effect of AAF, it did not alter participants’ tendency to overestimate errors when AAF was present. A third correlational study addressed whether effects of AAF are reduced for a subset of the population who may lack the kind of perception/action associations that lead to AAF disruption: poor-pitch singers. Effects of manipulations similar to those presented in Experiments 1 and 2 were reduced for these individuals. We propose that these results are consistent with the notion that AAF interference is based on associations between perception and action within a forward internal model of auditory-motor relationships.

## INTRODUCTION

Failures of error detection are obvious to anyone who has ever said the opposite of what they intended to say, only realizing their mistake once a friend points out the error. It is also possible to over-correct potential errors that may not occur, leading to dysfluencies in speech. Many factors can lead to errors, including errors of planning as well as factors related to the surrounding environment, including the stability of a device one controls (e.g., breakdown in the mechanics of a car while driving). Given the complexity involved in planning and execution during production, it is not surprising that the ability to detect errors is likewise error-prone and based on multiple factors. During a piano performance, for instance, demands primarily involve the rapidity and precision for the timing and sequencing of finger movements (Palmer, 1997). By contrast, during vocal performance the primary challenge involves continuous control of vocal pitch and adjustments to that pitch using laryngeal muscles (Sundberg, 1987).

Perhaps obviously, error detection relies to a great degree on the use of perceptual feedback from one’s actions. In the present research, we focus on auditory feedback in making and monitoring errors during music performance, incorporating both piano and singing production tasks. Auditory feedback is not the only source of feedback in music performance, and recent evidence suggests important roles for tactile feedback (e.g., Goebl and Palmer, 2008; Maidhof et al., 2013), and visual feedback (e.g., Kulpa and Pfordresher, 2013). Nevertheless, auditory feedback constitutes a good starting place, given that auditory events constitute the perceptual “goals” of music performance, and existing evidence suggests that motor planning is most strongly associated with such distal goals (e.g., Hommel et al., 2001).

Auditory feedback refers to the sounds that are associated with actions. It has been known for some time that altered auditory feedback (henceforth AAF), can severely disrupt production of action sequences, (for reviews, see Yates, 1963; Finney, 1999; Howell, 2004; Pfordresher, 2006), thus supporting the aforementioned link between auditory feedback and motor planning. Most research on AAF has addressed the role of synchronization of actions with sound. The present research incorporated alterations to feedback content (here, pitch associated with musical events), because such alterations more effectively simulate the errors one may produce. Moreover, the relationship between feedback content and planning is more well established than the relationship between feedback asynchrony and planning, given effects of asynchrony may simply reflect motor entrainment (Howell, 2001; Howell and Sackin, 2002; Pfordresher and Palmer, 2006; Pfordresher and Dalla Bella, 2011; Pfordresher et al., 2011; Pfordresher and Kulpa, 2011). Importantly, alterations of feedback content that disrupt production involve the presentation of events from within the sequence at the inappropriate serial position (e.g., hearing at every key press the pitch intended for the prior position), referred to as *serial shifts* of auditory feedback, whereas presentations of pitches unrelated to the sequence typically fail to disrupt (Finney, 1997; Pfordresher, 2005).

The disruptive effect of serial shifts is associated with the planning of serial order in production. Serial shifts resemble the kind of serial ordering errors pianists may make under normal conditions. Serial ordering errors often involve intrusions from nearby events, separated by distances of 1–4 events (in piano performance, Palmer and Pfordresher, 2003; Pfordresher et al., 2007; in speech, e.g., Vousden et al., 2000; in serial recall, e.g., Henson, 1998). Correspondingly, the most disruptive serial shifts involve the presentation of feedback events associated with these serial separations (Pfordresher, 2003; Pfordresher and Palmer, 2006). Thus, serial shifts appear to disrupt production because they involve presenting events to the participants that presently compete for production within the plan. Based on these similarities, serial shifts are thought to disrupt production because auditory feedback interferes with the planning of actions within a *shared representation* used to plan action sequences and to process auditory feedback (Pfordresher, 2006; cf. MacKay, 1987; Hommel et al., 2001; Hommel, 2009; Prinz et al., 2009).

An important corollary to the shared representation view described above is that altered feedback events may be mistakenly interpreted as originating from one’s action plan. Given that perception and action are based on a shared representation, AAF events can lead to confusions about their source and be mistaken for the intended consequences of actions, leading to an ambiguous experience of self-agency with respect to perception/action associations (e.g., Sato and Yasuda, 2005; Metcalfe and Greene, 2007; Couchman et al., 2012). As such, AAF events may be mistaken for errors of planning and/or execution. Other recent research is consistent with this view, but none to our knowledge has tested this proposal directly. A particularly important study comes from the domain of typing (Logan and Crump, 2010), which shares many features in common with piano performance (e.g., need to plan and order a complex sequence of motor responses, finger-key mapping is 1:many), while also differing in important ways (e.g., timing of responses not part of communicative goal). Logan and Crump (2010) had participants type single words on a computer and evaluate if they felt they typed it correctly or not. On some trials, the experimenters inserted errors into the words that the participants did not make, while on some other trials they displayed the correct word even if the participant misspelled it. Participants often assumed such feedback-based events were their own, superseding any knowledge of what they actually produced.

In the domain of music performance, another study assessed whether errors based on auditory feedback are treated as actual errors at a neural level (Maidhof et al., 2010). Maidhof et al. (2010) had pianists play bimanual keyboard sequences, and measured neural responses to errors committed by pianists as well as occasional feedback-based errors that were caused by shifting pitch of auditory feedback by a semitone on isolated events. Event-related potential (ERP) responses to both self-generated and feedback-based errors suggested some error-related negativity, though this negativity preceded self-generated errors but occurred after feedback-based errrors, leading to overall different ERP patterns. Given these differences in neural responses, it may be the case that feedback-based errors can be distinguished from self-generated errors the majority of the time. Other research suggests that the presence of auditory feedback in general may enhance error processing, as compared to performance with no auditory feedback (Herrojo-Ruiz et al., 2009).

Yet another corollary of the shared representation view is that individuals who have less well-formed associations between perception and action may show relatively less sensitivity to AAF, at least with respect to pitch content. We assessed this possibility by examining responses to AAF during singing for individuals varying in their ability to match pitch content in general. Recent evidence suggests that individuals who are deficient with respect to vocal pitch imitation (including, but not limited to singing) may be deficient with respect to the translation of perceived pitch into motor movements (Pfordresher and Brown, 2007). If AAF effects reflect interference within a shared representation of perception and action then, somewhat paradoxically, worse performing singers should also be less susceptible to disruption from AAF.

In addition to the role of auditory feedback in error processing, research reported here also addressed the role of executive functioning in the effects of auditory feedback on both production and error monitoring. Theoretical accounts of error monitoring can be found in the literature on cognitive control (e.g., Botvinick et al., 2001; Holroyd et al., 2005). These models, though differing in their details (specifically whether the core mechanism involves monitoring errors or sensitivity to response conflict), share the basic assumption that the detection of an error influences the allocation of attention within a task. If so, one may expect that processing of AAF involves executive control and as such a secondary executive task may reduce the influence of AAF on error monitoring (assuming that AAF effects are based on interpreting alterations as a production error). Another class of models that accounts for error monitoring is the internal model approach from motor control (e.g., Wolpert and Kawato, 1998; Hickok et al., 2011; Houde and Nagarajan, 2011). In contrast to cognitive control models, these models (described in more detail in the General Discussion, Section “Implications for the Underlying Shared Representation”) do not make claims about executive function.

In the present paper we report two new experimental studies that involve error monitoring during piano performance, and a correlational analysis of data from tasks that involve singing with AAF. In the two Experiments, we addressed whether AAF can occasionally be confused with committed errors during piano performance, and thus influence subjective estimates of error frequency. We manipulated AAF on an unpredictable number of events in a sequence (i.e., non-consecutively), so that AAF manipulations would not be too obvious and could in principle be mistaken for self-generated errors. In so doing, we were able to address a subsidiary research question. Past research using AAF of feedback pitch has either imposed the manipulation on a series of continuous events, or on single temporally isolated events (which tends not to disrupt production, e.g., Furuya and Soechting, 2010; Maidhof et al., 2010; Pfordresher and Kulpa, 2011). Pfordresher and Kulpa (2011) found increasing disruption from serially shifted feedback that varied with the number of altered events, but in that study alterations were all presented successively. We were thus able to address whether the successive presentation of AAF is a prerequisite for disruption.

## EXPERIMENT 1

The goals of the first experiment were to explore the influence of AAF on error monitoring. Participants (most non-musicians) performed a simple melody on a keyboard from memory in a cyclical fashion. We measured the frequencies of self-generated errors and compared these to estimated error frequencies made by participants after each trial. This task was performed with eight feedback conditions. Two control conditions comprised normal auditory feedback and a condition in which the keyboard was silent (auditory feedback was limited to mechanical sounds associated with key presses). The six other conditions involved AAF. One of these presented AAF on every successive event after a brief period of normal feedback (as is usually the case in past reports) and the remaining five conditions presented AAF on a subset of events.

It was hypothesized that AAF would produce more disruption than normal feedback (Pfordresher, 2003, 2005). Furthermore, if intermittent AAF produces the same effect found in Pfordresher and Kulpa (2011), then error frequencies should increase with the number of altered events. If error estimation accurately tracks error frequency, one should see an effect of feedback condition on error estimates that mirrors the one seen for error frequencies; however, if AAF disrupts error detection beyond its disruptive effect on production, then error estimates should differ from error frequencies. These hypotheses were tested by comparing the frequency of errors in a trial to the estimates that participants made at the end of a trial. Finally, comparisons across the normal and silent feedback conditions serve to test whether normal auditory feedback has any facilitative effect on performance.

### METHOD

#### Participants

Thirty-six students from the University at Buffalo participated in Experiment 1 in exchange for course credit. All but one reported being right-handed, and the mean age was 19.6 years (range: 18–32). Three participants reported experience playing the piano, with a mean duration of self-reported piano experience of 0.27 years and a range of 0–6 years^1^. All participants reported having normal hearing. Participants supplied informed consent, and all procedures reported here were carried out with approval of the Social and Behavioral Sciences Institutional Review Board at SUNY Buffalo.

#### Apparatus

Two experimental setups were used for this experiment, due to scheduling constraints in the lab. In one setup participants performed melodies on an M-AUDIO Keystation 49e unweighted-key piano, which was on a keyboard stand positioned at a comfortable height. Presentation of auditory feedback and metronome pulses as well as MIDI data acquisition was implemented using the FTAP software program (Finney, 2001) on a Linux operating system with millisecond resolution. Participants heard auditory feedback and metronome pulses over Sony MDR-7506 headphones at a comfortable listening level. The piano timbre originated from program #1 (Standard Concert Piano 1) of a Roland RD-700 digital piano. The second setup differed only with respect to the tone generator that was used. The piano timbre originated from program #1 (Standard Concert Piano 1) of a Roland SoundCanvas sc-55 mkII. Eight of the thirty-six participants completed the experiment on the second apparatus. Analyses incorporating setup as a factor yielded no effects related to the setup, and so this factor will not be considered further.

#### Materials and conditions

**FIGURE 1:**
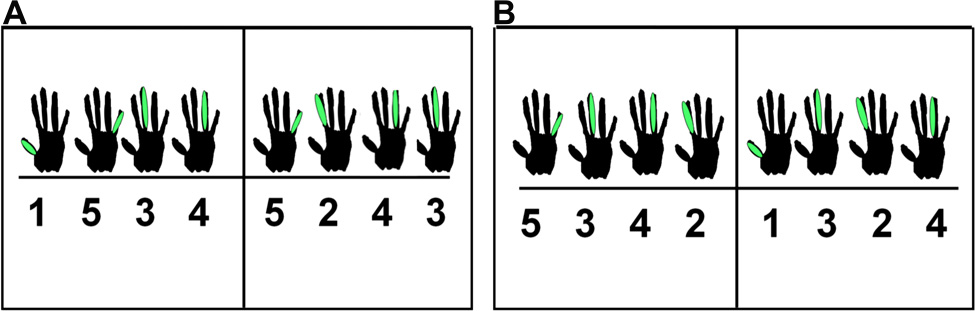
**Notation used for melody 1 (A) and melody 2 (B)**.

Participants played five immediately consecutive repetitions of an eight-note melody in a trial, leading to 40 produced (and feedback) events. The two melodies that participants could play are shown in **Figure 1**, using notation designed for non-pianists. Each of these melodies was performed on the white piano keys C4–G4 with the participant’s right hand. The first melody’s sequence of notes was C-G-E-F-G-D-F-E, and the second melody’s sequence of notes was G-E-F-D-C-E-D-F. Participants learned and rehearsed one melody during a practice session and played that melody on every trial of the experiment. Analyses incorporating stimulus melody as a factor yielded no effects related to melody, and this factor will not be considered further.

Alterations to auditory feedback were determined in the following way. In all AAF trials the first eight events (the first repetition of the melody) remained unaltered to ensure that participants had a chance to become accustomed to the melody. Positions of altered events in a trial were established within a parameter file set up in FTAP, choosing from the remaining 32 sequence positions. For trials in which all events were altered (comparable to most earlier studies on the effects of AAF), every event from position 9 through 40 was altered with respect to pitch. For trials with intermittent AAF, the events chosen to be altered were the first *x* items of a random permutation of integers 1 through 32, with *x* being the number of altered events according to the feedback condition. This process was repeated five times to give each condition five different parameter files so that each condition had five different permutations of altered sequence events. In essence, each feedback condition had five different randomly selected sets of altered notes, and each participant experienced all five sets. **Tables 1** and **2** describe the properties of altered events in the parameter files of the five conditions of intermittent AAF.

**Table 1:** Frequency of altered events at sequence positions associated with different categories of metrical accent, contour accent, and tonal pitch class.

# of AAF events	Metrical accents Both melodies		Contour accents Both melodies		Tonal pitch class First melody		Tonal pitch class Second melody	
	Strong	Weak	Change	Same	Strong	Weak	Strong	Weak
2 events	3 (30%)	7 (70%)	8 (80%)	2 (20%)	6 (60%)	4 (40%)	6 (60%)	4 (40%)
4 events	12 (60%)	8 (40%)	13 (65%)	7 (35%)	17 (85%)	3 (15%)	12 (60%)	8 (40%)
8 events	16 (40%)	24 (60%)	33 (83%)	7 (17%)	26 (65%)	14 (35%)	21 (53%)	19 (47%)
16 events	42 (53%)	38 (47%)	59 (74%)	21 (26%)	52 (65%)	28 (35%)	42 (53%)	38 (47%)
24 events	58 (48%)	62 (52%)	90 (75%)	30 (25%)	77 (64%)	43 (36%)	67 (56%)	53 (44%)

*Frequencies associated with metrical accents and contour accents did not differ between the two melodies; the position of tonal pitch classes did differ, so each melody has its own column. Strong metrical accents occur at positions 1 and 3 in each bar, and strong tonal pitch classes include the tonic, third, and fifth.*

**Table 2:** Distribution of altered events by position in the sequence, summed across the five parameter files for each condition.

# of AAF events	First iteration	Second iteration	Third iteration	Fourth iteration	Repeat altered positions
2 events	3 (30%)	3 (30%)	2 (20%)	2 (20%)	1 (2.5%)
4 events	5 (25%)	11 (55%)	3 (15%)	1 (5%)	5 (10%)
8 events	11 (28%)	12 (30%)	6 (15%)	11 (27%)	10 (25%)
16 events	19 (24%)	22 (27%)	20 (25%)	19 (24%)	25 (63%)
24 events	30 (25%)	29 (24%)	25 (21%)	35 (30%)	30 (75%)

*Iteration refers to a single repetition of the eight-note melody. Thus, altered events can fall in one of the four iterations that contain the 32 notes of the performance eligible for alteration. “Repeat altered positions” refers to the number of positions in the sequence that are altered in at least two parameter files.*

**FIGURE 2:**
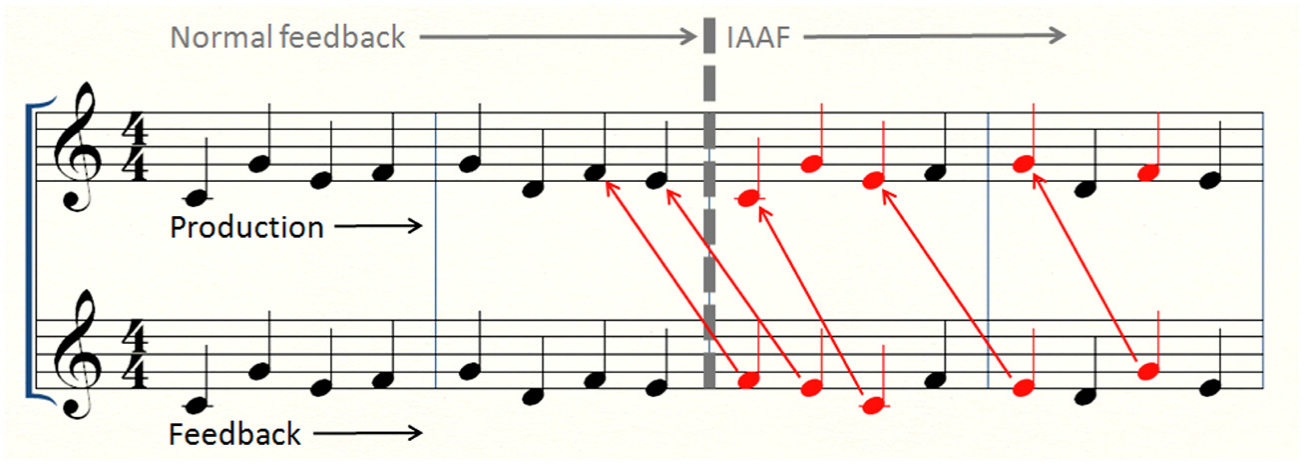
**Music notation for the first two repetitions (out of five) of melody 1.** The top row displays production, the bottom row displays feedback, and the gray barrier indicates the transition from normal feedback to 16-event AAF. Red notes are altered to a lag-2 serial shift of pitch.

Pitches were altered to match the pitch performed two key presses prior to the current event. This manipulation constitutes a *serial shift* of lag-2. An example of the effect this alteration has on performance for a trial with intermittent AAF can be seen in **Figure 2**, which depicts the music notation of what a participant would hear when playing an accurate performance of melody 1 while listening to 16-event AAF. We chose the lag-2 serial shift for several reasons. First, in pilot studies, we found that altering pitch events to match a randomly selected pitch failed to disrupt production, in keeping with other research on altered feedback effects (Finney, 1997; Pfordresher, 2005). Second, errors that occur in performance with normal auditory feedback often involve target-intruder relationships separated by distances of two events (Palmer and Pfordresher, 2003; Pfordresher et al., 2007). Finally, if we had used a lag-1 serial shift (the most common manipulation in past experiments), this manipulation would have caused frequent repeated feedback pitches in trials with intermittent AAF.

#### Design

The primary independent variable for Experiment 1 was the number of altered feedback events. This was a within-subjects variable with eight levels. On a given experimental trial the participant might hear no auditory feedback, normal auditory feedback, or serially shifted auditory feedback on 2, 4, 8, 16, 24, or 32 events (0, 6.25, 12.5, 25, 50, 75, and 100% of the feedback events eligible for alteration). Conditions were presented to participants in one of two predetermined, pseudo-random orders (a between-subjects variable); in each of these predetermined orders, participants always experienced different feedback conditions on successive trials, and the conditions cycled such that the participant experienced each feedback condition once by the end of every eight trials. There were 10 such cycles in an experiment; thus participants who completed all trials (all except 4) performed 10 repetitions of each AAF condition^2^. Participants were also assigned to play one of two melodies in the experiment (19 melody 1, 17 melody 2) and this melody assignment functioned as a second between-subjects variable.

#### Procedure

Participants were asked to complete a piano experience questionnaire prior to starting the experiment. After giving their consent to participate in the study, participants were given as much time as was needed to learn the melody, using notation shown in **Figure 1**. The learning phase was over when participants reported feeling comfortable enough to play the melody in the absence of the notation. We confirmed their memorization by having them produce three error-free repetitions of the melody.

Experimental trials followed. The experimenter told participants that they would perform the melody they had been assigned in the practice phase. Participants were informed that auditory feedback might not always match what they expected to hear. Participants were encouraged to continue to play to the best of their ability and to keep playing at the same tempo even if AAF disrupted their performance. Participants were also told that at the end of each trial, the experimenter would ask them how many errors they thought they made. It was emphasized to the participant that “errors” in this case meant, “wrong key presses, not necessarily wrong sounds.” Instructions thus generally encouraged participants to ignore auditory feedback.

To summarize, each experimental trial had the following structure. Participants would perform five repetitions of the eight-note melody, ending their performance on a sixth repetition of the first note so the melody would resolve. On the second through fifth repetitions in a trial, altered feedback was implemented for a variable number of key presses, except in the normal feedback condition, which had no alterations. During the silent condition, feedback was removed after the first repetition. After performing the melody, participants reported aloud to the experimenter the number of errors they thought they made. Participants took a short break halfway through the experiment during which they filled out questionnaires asking them about their background in language, music, and hearing.

The entire session lasted approximately 1 h.

#### Data analysis

There were two dependent variables of interest. The first dependent variable was the number of errors made by the participant on each performance. This variable was measured by calculating the fewest number of changes that would need to be made to the participant’s performance to match an ideal performance, using error processing algorithms that are commonly used for music performance (Large, 1993; Palmer and van de Sande, 1993, 1995). The second dependent variable was the number of errors that the participant estimated on each performance. We report each count as a percent of the total number of events produced in a trial (40). This variable was gathered by self-report and recorded by the experimenter. From these variables we computed a third variable of interest which was the difference between estimated and produced errors, structured so that positive values indicate an overestimation of error frequency. We also gathered data on the speed and consistency of participants’ performances for each trial, as measured by the mean inter-onset interval (average across all participants and trials = 360 ms) and its coefficient of variance (M/SD, average across participants and trials = 0.20). In the interest of brevity, however, we focus here on error rates given their conceptual importance to the question at hand^3^.

Means for error frequencies, error estimates, and difference scores were put into a one-way ANOVA with the factor feedback condition. ANOVA results were submitted to two further analyses. First, Dunnett’s tests were used to determine which conditions differed from the normal feedback control condition (including each AAF condition and the silent condition). Second, a linear trend test was conducted across the six AAF conditions (excluding the normal and silent control conditions), to determine whether increasing the frequency of AAF led to comparable increases in any dependent measure. For difference scores, we also ran *t*-tests within each condition to determine if the mean differed significantly from zero (indicating over or under-estimation). We report standard ANOVA results from untransformed data. However, it is worth noting that all analyses reported here as significant remain significant after applying the Greenhouse–Geisser correction for violations of sphericity, after applying the arcsine square-root transformation, and if the nonparametric Friedman’s test is used instead of ANOVA.

### RESULTS

#### Error frequency

**FIGURE 3:**
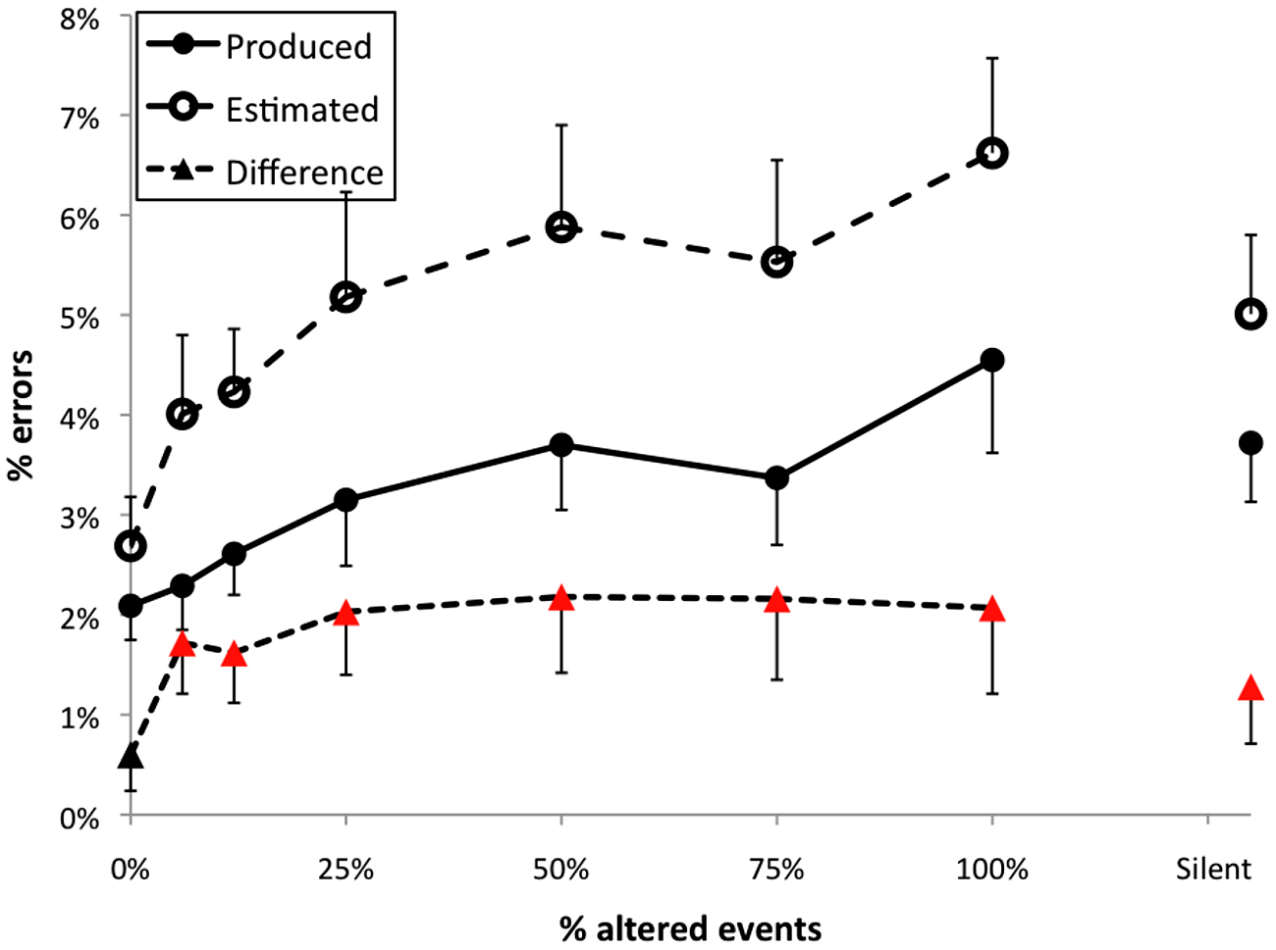
**Results from Experiment 1.** Error bars indicate 1 SE of the mean; triangles plotted as red higlight those conditions for which the difference between produced and estimated error frequencies differed significantly from zero.

The percent of incorrectly performed pitch events in Experiment 1 across feedback conditions is shown in **Figure 3** (filled circles). The feedback manipulation had a significant effect on the number of errors made by participants, *F*(7,245) = 4.51, *p* < 0.001. The Dunnett’s test revealed that errors per trial were significantly different from normal feedback when 50, 75, or 100% events were altered. Somewhat surprisingly, errors in the silent feedback condition were also significantly greater than during normal feedback, in contrast to results of several previous studies (Finney, 1997; Finney and Palmer, 2003; Pfordresher, 2005). Within altered feedback conditions there was a significant linear trend, *t*(35) = 2.82, *p* < 0.01, indicating that disruption increased in proportion to the number of altered events.

#### Error estimates

The mean percent of errors estimated by the participants for each altered feedback condition is shown by open circles in **Figure 3**. The feedback manipulation had a significant effect on the number of errors estimated, *F*(7,231) = 11.89, *p* < 0.001. The Dunnett’s test revealed that mean error estimates in all feedback conditions except 6.25% (two-events altered) intermittent AAF were significantly different than error estimates made under normal feedback, and estimated errors in the silent feedback conditional also significantly exceeded normal feedback. Within altered feedback conditions there was a significant linear trend, *t*(35) = 5.73, *p* < 0.001, indicating that estimates of error frequency increased in proportion to the number of altered events.

#### Difference scores

Differences between estimated and self-generated error frequencies were also calculated for each trial, as shown in **Figure 3** (filled triangles). Feedback had a significant effect on the difference between the frequency of errors and participants’ self-report *F*(7,245) = 3.28, *p* < 0.01. The Dunnett’s test revealed higher difference scores than the normal feedback condition for conditions with 6.25, 25, 50, 75, or 100% of events altered, but no difference between the normal and silent feedback conditions. However, the linear trend test on difference scores was non-significant (*p* = 0.20). In addition, difference scores exceeded zero in all conditions other than the normal feedback condition (highlighted using red in **Figure 3**). AAF thus led to significant overestimation of error rates (with some overestimation present for silent feedback).

**FIGURE 4:**
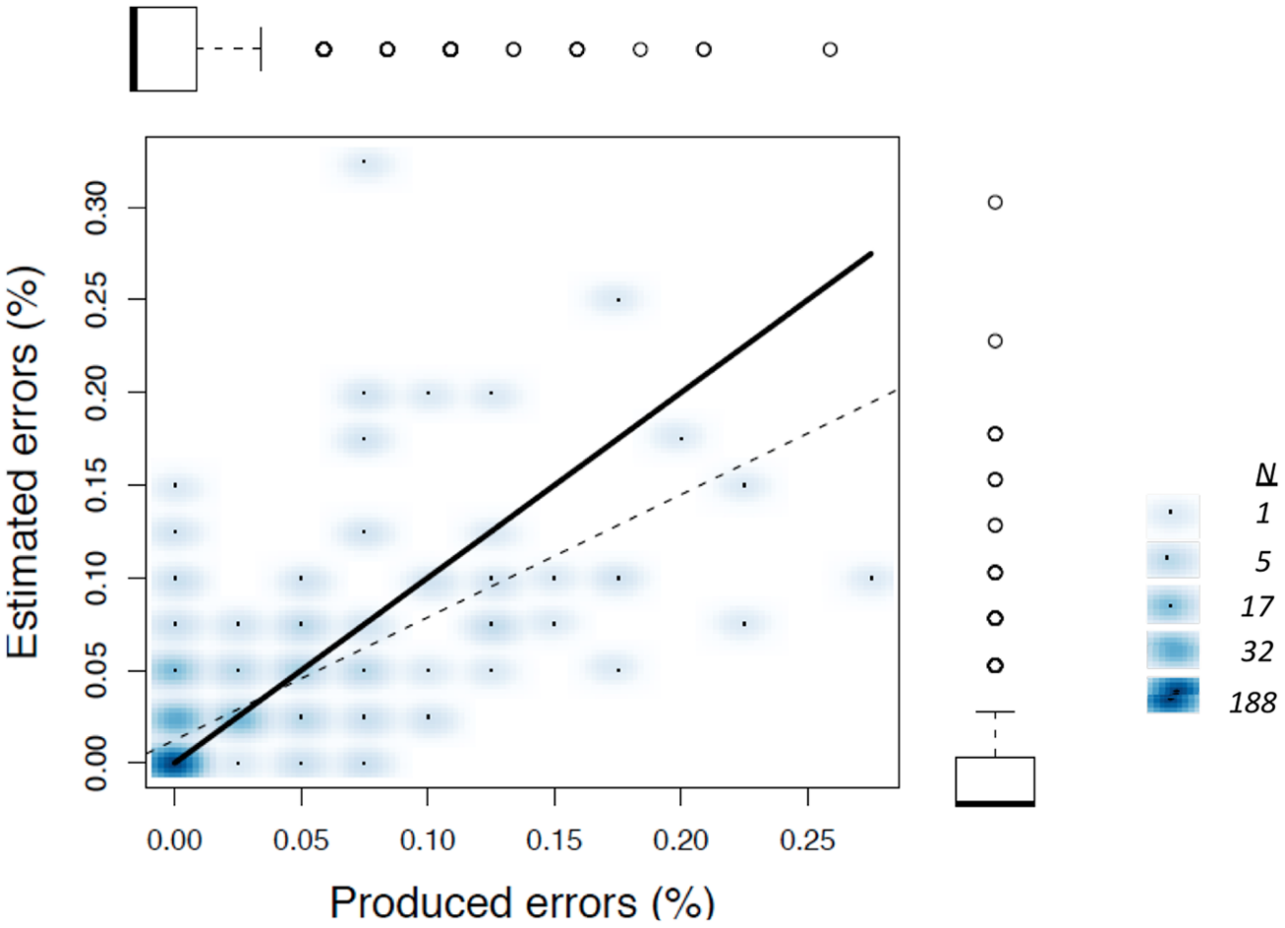
**Smoothed scatterplot showing the relationship beween produed and estimated errors within the normal feedback condition from Experiment 1.** Boxplots aligned with each axis show frequency distributions, with outliers shown as open circles. Estimates from all individual trials are plotted (*N* = 352). Unity is shown by the dark line, and the best-fitting least squared regression is shown by the dashed line. The legend on the right highlights frequency counts associated with selected shades.

We were initially concerned that the tendency to overestimate error frequencies during AAF trials may simply occur because participants made more errors during these trials, making number estimation more error-prone than when participants made few errors (cf. Whalen et al., 1999). If overestimation during AAF trials is a byproduct of error frequency, then one might expect that a regression of estimated on actual error frequency within the normal feedback trials would have a slope greater than one, with overestimations occurring when participants happened to make more errors even without AAF. This was not the case, as shown in **Figure 4**. Because the frequency of individual observations varies considerably, due to the fact that participants were estimating integer values, the scatterplot shown here uses shading to indicate differences in frequency for each data point (using the “smoothed scatterplot” function in R). The regression accounted for a significant proportion of variance in estimated error frequencies (*r*^2^ = 0.41), with a slope considerably lower than one, *B* = 0.662.

### DISCUSSION

Experiment 1 found significant effects of AAF on both self-generated error frequencies and estimates of error frequencies. Both variables increased in proportion to the frequency of AAF in a trial. In addition, difference scores between estimates and self-generated errors suggest that overestimation of error frequencies occurred in all conditions except for normal feedback. The tendency to overestimate errors was not simply based on error frequency. However, the overestimation effect is slight in comparison to the number of events that could be interpreted as errors (i.e., altered feedback events). This suggests, first, that participants are not simply counting AAF events to estimate errors, and second, that any possible illusion of authorship, in which altered feedback is falsely identified as an error (cf. Logan and Crump, 2010), occurs occasionally. Error frequencies would be much higher if the illusion of authorship happened on every altered event, resulting in error frequencies that approximate the number of altered events rather than the number of errors.

## EXPERIMENT 2

In Experiment 2 we investigated the relationship between cognitive load and error detection. As mentioned in the introduction, many models of error detection assume that this process involves executive resources. However, it is not clear whether error monitoring within the production of rapid, well-learned sequences involves such functioning. If monitoring of auditory feedback requires executive functioning, one would expect the overestimation tendency to diminish when a cognitive load task is introduced. In addition, the introduction of the executive load task allowed use to evaluate whether the use of executive resources, which also may play a role in planning of sequences, makes performers more vulnerable to the effect of AAF. Thus, we were interested in whether cognitive load influenced disruption from AAF (related to use of executive resources in production) as well as the overestimation tendency (use of executive resources in error monitoring).

In varying cognitive load, we incorporated the digit-probe task from Milton et al. (2008), whose participants categorized line drawings of boats while remembering a six-item string of digits. This particular task was chosen due to the fact that it involves memory for serial order, which is also necessary for the production of musical sequences. Thus it is likely to involve the sort of executive resources that are relevant to the task at hand.

### METHOD

#### Participants

Because this experiment took 50% longer to complete than Experiment 1, fewer participants could complete the task. Twenty-two students from the University at Buffalo participated in exchange for course credit. All but one reported being right-handed, and the mean age was 19.8 years (range: 18–25). Ten participants reported experience playing the piano; mean duration of self-reported piano experience was 2.14 years with a range of 0–19 years of experience. An additional four participants had memory of playing casually. All participants reported normal hearing.

#### Materials

Experiment 2 used the same materials as Experiment 1, but it also used a list of six-digit numbers that were randomly generated. The same list was used for all participants, but the items in the list were presented in a different order for half of the participants. The exact procedure of the task was changed slightly to meet our equipment constraints. Each of the six digits in the sequence was read aloud to the participant at an approximate 500-ms IOI (Milton et al., 2008, had an automated voice read the digits at 330-ms IOI), with approximately 2 s between the last item and the start of the metronome. Approximate values are given because the experimenter read the digits aloud at the prescribed rate by remembering the tempo of the metronome from practice trials and previous experimental trials.

The following limitations were placed on the selection of the 80 six-digit sequences. The digits 0–9 occurred only once in a given sequence; ascending or descending patterns of adjacent digits were not allowed to go on for more than two items in a sequence (e.g., 231 is allowed but 234 is not); a digit cannot occupy the same position in two adjacent sequences (e.g., if sequence A ends in 0, then sequence B cannot end in 0).

#### Design

The primary independent variable from Experiment 1, feedback condition, was crossed with the presence or absence of the load task in the trial, resulting in a 2 (presence of load) × 8 (feedback condition) within-subjects design. As in the Experiment 1, the conditions were presented to participants in a pseudo-random order, and each participant was assigned to one of two order conditions that determined the sequence of this presentation and one of two melodies (12 played melody 1, 10 melody 2). The presentation order of the six-digit sequences was determined by this condition as well. We presented fewer repetitions of each trial type than in Experiment 1 (5 rather than 10), in order to maintain the 1-h timeframe while still having a sufficient number of trial repetitions to get reliable accuracy data.

#### Procedure

The procedure in Experiment 2 was nearly identical to Experiment 1 with the following exceptions.

First, the participant was informed of the presence of the load task during the instructions given during the consent process. The load task was described to the participant as a “memory test” that would be given on certain trials to make those trials more challenging.

Second, after the participant demonstrated their ability to perform the melody without the help of the visual aid, the experimenter (who was the second author) coached the participant through a practice round of the load task. The experimenter would say, “I’m going to read you a list of numbers, and I want you to try to remember them. While you’re keeping those numbers in your head, I want you to play through the melody like you just did. Then, I’m going to ask you about one of the numbers in the list I read to you. Are you ready?” When the participant indicated he or she was ready, the experimenter read the numbers aloud, paused for a brief waiting period, and then began the metronome. Upon completion of the trial, the participant was asked, “What number came after ____?” After hearing the participant’s response, the experimenter gave the participant encouraging feedback regardless of whether their answer was correct or not. Estimates of error frequency (carried out as in Experiment 1) were collected after participants responded to the load task. On a control trial, the participant was told, “There are no numbers to remember on this trial,” prior to the start of the trial. The trial then proceeded identically to Experiment 1.

#### Data analysis

Data analyses were carried out as in Experiment 1. For the purpose of Dunnett’s test comparisons and linear trend tests, we separated the two load conditions and computed error terms separately within each condition^4^ .

### RESULTS

#### Error frequency

**FIGURE 5:**
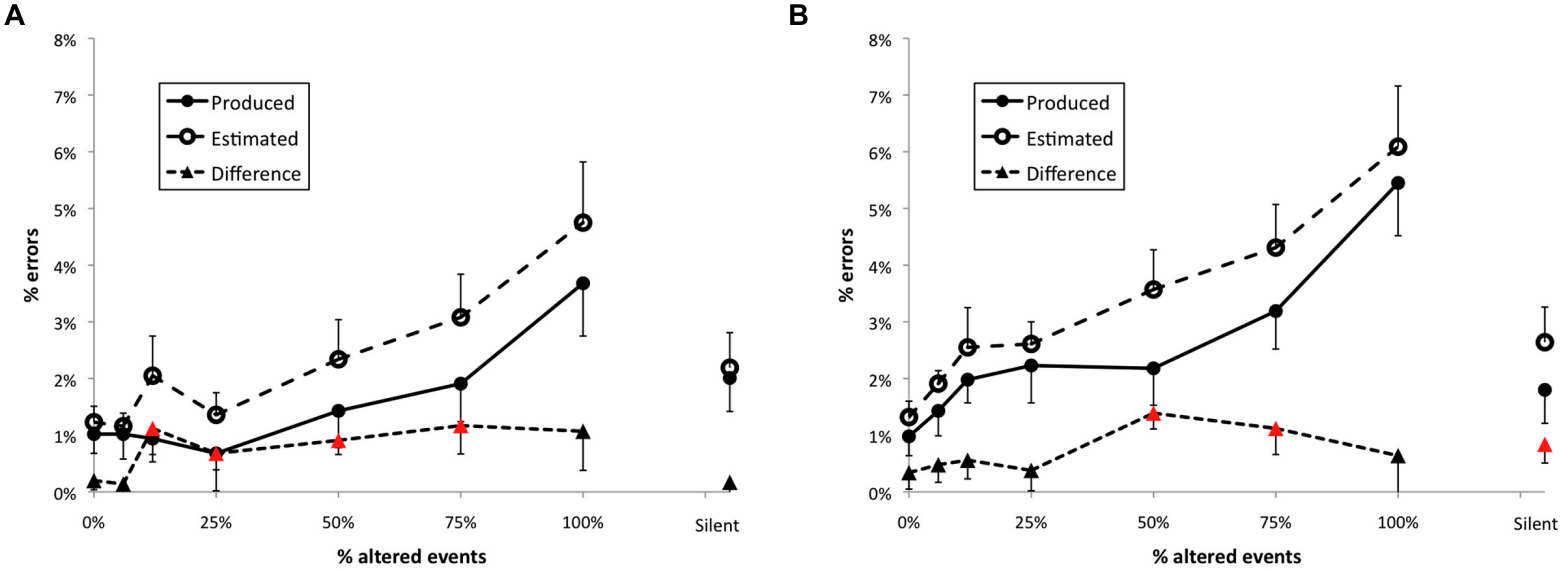
**Mean percent of errors per trial plotted by feedback condition for Experiment 2 for trials with no load (A) and with the load task (B).** Error bars are equal to 1 SE of the mean; triangles plotted as red higlight those conditions for which the difference between produced and estimated error frequencies differed significantly from zero.

The percent of produced errors across feedback conditions is shown in **Figure 5** for trials without the load task (**Figure 5A**) and with the load task (**Figure 5B**). The feedback manipulation had a significant effect on the number of errors made by participants across load conditions, leading to a significant main effect, *F*(7,147) = 9.99, *p* < 0.001. There was also a main effect of cognitive load on the frequency of errors, *F*(1,21) = 13.44, *p* < 0.001. Participants made more errors per trial when they had to do the load task concurrently with the performance than without it. Additionally, there was a significant load × feedback interaction, *F*(7,147) = 2.26, *p* < 0.05, supporting the prediction that the effect of AAF on errors was accelerated when the load task was present compared with the no-load condition.

The effect of AAF within each load condition was further addressed by analytical comparisons using Dunnett’s test. In each of the load conditions error frequencies increased relative to normal feedback when 75 or 100% of events were altered. Furthermore, both load conditions exhibited a significant linear trend within altered feedback conditions [no load, *t*(21) = 2.82, *p* < 0.01, load, *t*(21) = 4.66, *p* < 0.001].

#### Error estimates

The feedback manipulation had a significant effect on the number of errors estimated by the participants, *F*(7,147) = 4.28, *p* < 0.01. There was also a significant main effect of load, *F*(1,21) = 25.87, *p* < 0.001, but no interaction (*p* = 0.601). As was true for produced errors, Dunnet’s tests on estimated errors indicated increased estimates, relative to normal feedback, when 75 or 100% of events were altered in each load condition. An additional significant contrast in the load condition was found when 50% of events were altered. Both load conditions exhibited a significant linear trend within altered feedback conditions [no load, *t*(21) = 3.08, *p* < 0.01, load, *t*(21) = 4.88, *p* < 0.001].

#### Difference scores

Surprisingly, the two-way ANOVA and linear trend test on difference scores yielded no significant effects. However, while the difference scores failed to vary by feedback condition, several were significantly different from zero, continuing the pattern of overestimation observed in Experiment 1, as shown by red triangles.

#### Load task accuracy

**FIGURE 6:**
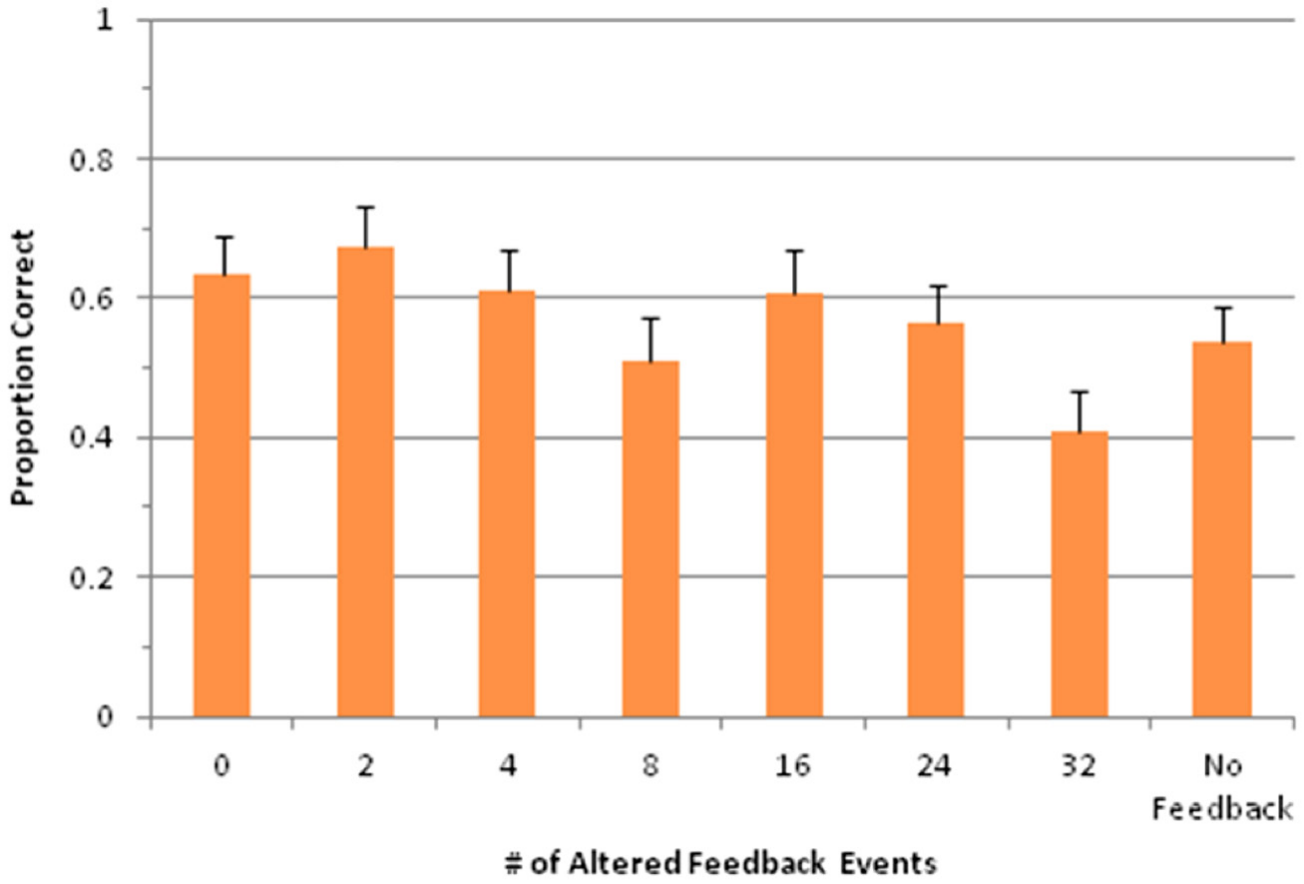
**Proportion of correct answers in the load task for each feedback condition.** Error bars represent 1 SE of the mean.

An additional consideration for Experiment 2 was whether performance on the load task differed as a function of feedback condition. **Figure 6** shows performance on this task across all conditions. Overall accuracy for the load task was 56.9%, which was well above chance (17%), and this was true for each individual condition (*p* < 0.001 for each condition, single-sample *t*-test). An ANOVA revealed a significant main effect of feedback condition on load task accuracy, *F*(7,147) = 3.45, *p* < 0.01. Importantly, this effect related to significantly lower accuracy for AAF conditions that also led to more production errors, as can be seen in **Figure 6**. Thus, participants did not trade off one task for the other.

### DISCUSSION

Experiment 2 investigated whether taxing executive resources would produce more disruption in music performance than if those resources were not taxed. In line with our prediction, concurrently remembering a sequence of numbers is associated with greater overall disruption of performance (more errors) as well as an exacerbated disruption for trials with a high proportion of altered feedback events. Concurrently remembering those numbers is also associated with greater overall error estimates; however, the elevated error estimates produced by altered feedback are equal in the presence and absence of the cognitive load. It may be the case that cognitive load makes participants more vulnerable to errors from AAF because load interferes with planning resources that participants rely on to maintain accurate performance when feedback is disruptive. However, the effect of AAF on error estimation seems not to be affected by cognitive load, suggesting that the error monitoring process may be automatic.

Mean accuracy on the load task was 56.9%, which is much lower than the 76.4% accuracy rate found by Milton et al. (2008). This discrepancy may have occurred because the music performance task used here is more difficult to accomplish than the sorting task conducted by Milton and colleagues. An alternative but not mutually exclusive explanation is that participants may have chosen to pay more attention to their musical performance than remembering the numbers, resulting in lower accuracy. Finally, the difference in inter-test-interval may have been a contributor as well. The temporal separation between the presentation of the numbers and their probing was 1.5 s in the study of Milton and colleagues whereas in the present study the separation was around 20 s, depending on the speed of the performer. Such a delay likely contributed to decay in working memory.

## AAF EFFECTS ON SINGING

Next, we present a correlational analysis concerning AAF effects similar to those reported above for a different music performance task: Singing. Previous research has shown similar disruptive effects of AAF manipulations for keyboard performance and singing, with larger effect sizes for singing when participants hear altered pitch events (Pfordresher and Mantell, 2012). The present analysis addresses the shared representation hypothesis, as did the two piano performance experiments, but from a different perspective. Rather than address whether AAF influences error monitoring, we now address individual differences in the disruptive effect of AAF when pitches are altered. Specifically we are interested in whether individuals who seem to lack a properly functioning shared representation – poor-pitch singers – also exhibit reduced susceptibility of AAF.

Poor-pitch singing is a deficit of vocal pitch imitation leading to persistent shifting of imitated pitch by more than a semitone. Current estimates suggest that this deficit appears in a minority of the population, though much more frequently than deficits of perception (Dalla Bella et al., 2007; Pfordresher and Brown, 2007; Pfordresher et al., 2010; Hutchins and Peretz, 2012). As such, most poor singing is probably not based on perceptual deficits, and is likely to result from a deficit of sensorimotor translation (Dalla Bella et al., 2007; Pfordresher and Brown, 2007; Hutchins and Peretz, 2012). This deficit of translation may be based on a deficient shared representation, which integrates action plans with perceptual events. Furthermore, poor-pitch singing is a deficit that appears to be specific to pitch and not to the imitation of timing (Dalla Bella et al., 2007).

We addressed this hypothesis by pooling data from two experiments that involved singing with AAF, but that were not originally designed to address individual differences (Pruitt and Pfordresher, 2011; Pfordresher and Mantell, 2012). In these experiments, participants sang melodies like those used in Experiments 1 and 2 while hearing normal feedback, serially shifted pitch content (of lag-1), or slight asynchronies that disrupt timing of production but that do not typically disrupt accuracy of pitch sequencing (Pfordresher, 2003, 2006). Given the predictions stated above, we predict that participants who show overall lower accuracy at pitch imitation (singing in tune) should show reduced disruption from AAF that affects pitch. By contrast, we predicted that a similar reduction would not be found for AAF manipulations of feedback synchrony, which are presumed to disrupt production based on internal timekeeping mechanisms rather than a forward model relating action planning to the pitch content of feedback (cf. Pfordresher, 2011).

**FIGURE 7:**
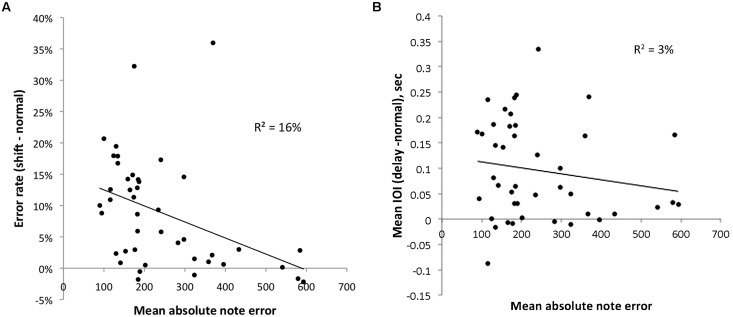
**Relationship between degree of mistuning for individual sung pitches (X-axis), and the disruptive effects of altered auditory feedback (Y-axis).** Different panels incorporate Y-axes that represent the disruptive effects of serially shifted feedback on production errors **(A)** and the disruptive effect of asynchronous feedback on production rate **(B)**.

**Figure 7A** plots the relationship between mean absolute note error, the average absolute difference between target and imitated pitch on all trials of an experiment (a measure of vocal imitation accuracy) in cents (100 cents = 1 semitone), and the disruptive effect of lag-1 serial shifts in singing tasks like those used here. Although values on the X and Y-axes come from the same data set they were analyzed differently. Critically, Y-axis values were based on errors of melodic contour (direction of pitch changes), whereas X-axis values were based on the degree of “mistuning” found for individual sung pitches.

As can be seen, the relationship between these variables is negative, suggesting that participants who exhibit larger levels of mistuning show less disruption from alterations to auditory feedback. The relationship was statistically significant, *r*(42) = -0.40, *p* < 0.01. This correlational analysis supports our hypothesis that individuals with poorly functioning internal models do not rely on perceptual feedback as much as more accurate singers.

As mentioned earlier, we also assessed the disruptive effects of alterations to the synchrony of auditory feedback across individuals. This analysis is important because it is possible that poor singers are simply less attentive to auditory feedback and that the reduction in disruption found here could be found for AAF relationships that presumably do not influence the same kind of sensorimotor associations for pitch. In this context, the results shown in **Figure 7B** are informative. **Figure 7B** shows an analysis similar to what is shown in **Figure 7A**, except that the Y-axis represents the disruptive effect of asynchronous (delayed) auditory feedback on timing in production. As can be seen, the relationship is much weaker, and not statistically significant. In addition to providing a convenient control, this result is also consistent with aforementioned evidence that poor singing is specific to the pitch dimension (e.g., Dalla Bella et al., 2007), and is consistent with recent evidence suggesting that asynchronous AAF influences a distinct neural network from pitch alterations (Pfordresher et al., 2014).

## GENERAL DISCUSSION

The purpose of the current work was to illuminate the relationship between error monitoring and altered feedback. In the two new experiments we report, AAF led participants to overestimate the frequency of produced errors. Furthermore, the introduction of a load task increased disruption but did not increase the overestimation tendency (which in fact was somewhat smaller in Experiment 2 than Experiment 1). Finally, a correlational analysis of singing data suggest that individuals who lack vocal-motor associations for pitch (poor-pitch singers) are less responsive to AAF manipulations of pitch content. We here reflect on the significance of these findings.

### IMPLICATIONS FOR THE UNDERLYING SHARED REPRESENTATION

A general theme connecting the results reported here has to do with the hypothesis that AAF disruption occurs because perception and action share a common representation, and AAF therefore disrupts action planning because it adds activation to events planned for alternate sequence positions (Pfordresher, 2006). The studies here support this view, but also point to a specific kind of model that may relate to AAF effects.

A particularly informative finding comes from the effects of cognitive load in Experiment 2. Here, error frequencies and error estimates both increased under the concurrent load task. However, the load task did not increase the tendency to overestimate errors in the presence of AAF. These results suggest that motor planning may require some use of executive control, but (somewhat paradoxically) that the relative balance of feed-forward (i.e., motor based) and feedback information in error monitoring is not influenced by the availability of executive resources. Thus, the error monitoring processes here may have less in common with models of cognitive control used in decision-making tasks like flanker interference (e.g., Holroyd et al., 2005), and may be better explained within a motor-control framework. A framework that we propose may be amenable to the present results is that of the internal model.

The internal models framework mentioned in the introduction has gained increasing prominence as a way of accounting for sensorimotor integration in tasks ranging from grasp control (Kawato, 1999) to speech disorders (Max et al., 2004). The basic logic of the internal model construct is that motor planning incorporates internalized knowledge about how actions are related to their perceptual effects (via a sensorimotor model in the brain). It has been proposed that internal models can be instantiated to serve two purposes: *Inverse* models use the internal model to plan actions based on the anticipated outcome of those actions. By contrast, *forward* models use the internal model to gage the outcome of a planned action in advance of perceptual feedback from that action. Forward models have played a major role in the literature as accounts that address the fact that motor errors can be corrected more rapidly than would be possible if one were to use perceptual feedback. We suggest that the results found here are best accounted for by the forward model construct.

**FIGURE 8:**
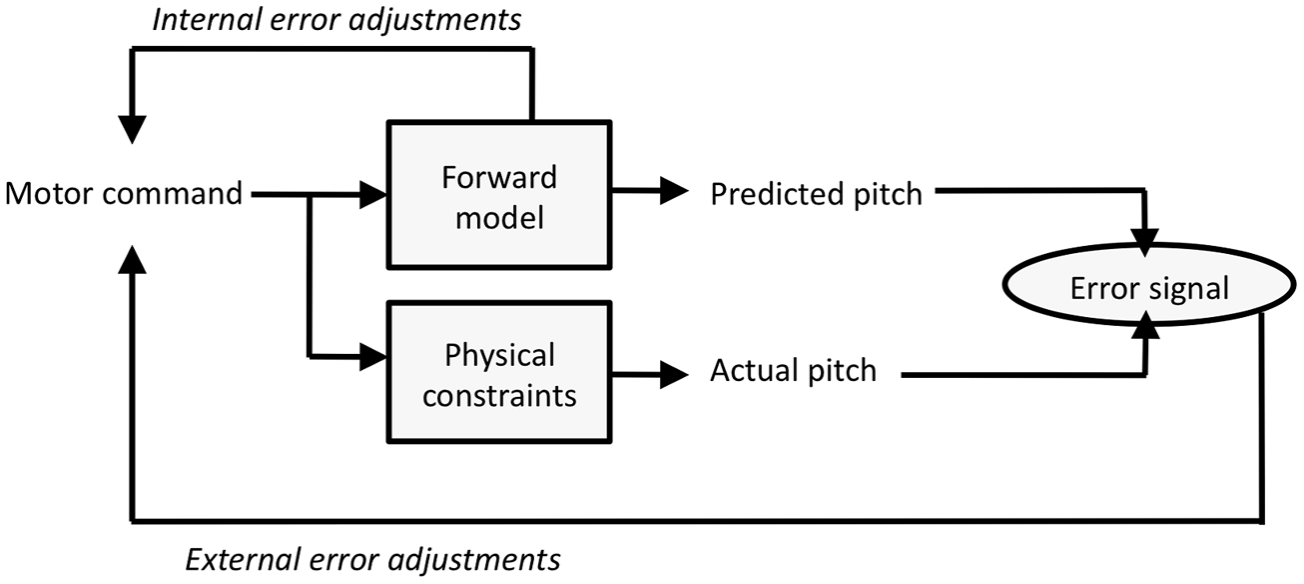
**A simplified forward model framework adapted to the present task**.

**Figure 8** presents a simplified forward model framework that is adapted to the present experimental context (cf. Jordan and Rumelhart, 1992; Wolpert and Kawato, 1998; Houde and Nagarajan, 2011). The motor command (a finger movement or level of vocal fold tension) feeds into the forward model to generate an anticipated pitch outcome (this outcome can be used for internal error correction). In addition, the motor command leads to an actual pitch event, which can be influenced by current physical constraints which here includes possible artificial alterations of events. Differences between the anticipated and actual pitch outcomes yields an error signal, which in turn generates adjustments to the motor command. Typically, errors are attributed to problems of prediction in the forward model and lead to adaptation effects (e.g., Houde and Jordan, 1998, 2002). What happens in the present paradigm is that the physical constraints occasionally lead to pitches that differ from predictions, which may occasionally be misattributed as motor command errors.

With respect to singing, earlier research has suggested that poor singers lack a properly functioning *inverse* internal model of auditory–vocal relationships (Pfordresher, 2011) but it has not yet been clear whether this deficit generalized also to forward modeling. Evidence for an inverse model deficit stems from several recent studies. For instance, poor singers report reduced ability to imagine melodies and other auditory events (Pfordresher and Halpern, 2013), suggesting that poor singers lack the sensorimotor associations that underlie auditory imagery (e.g., Halpern, 2001). Other recent evidence suggests that poor-pitch singing deficits are reduced when singers imitate recordings of themselves, in keeping with the notion that inverse models allow one to imitate perceptual events that have not been performed in the past (Pfordresher and Mantell, 2014). The present data suggest that poor singing deficits may not be limited to inverse modeling, but may also involve the forward model component, given that poor singers respond differently to perceptual feedback.

### AUDITORY FEEDBACK AS AN “ERROR SIGNAL”

Early studies on the effects of altered feedback assumed that disruption occurred because altered feedback is interpreted as an error signal (e.g., Lee, 1950; Black, 1951). According to this view, auditory feedback when altered informs the action plan that an error has been made, thus leading to adjustments to the plan that are inappropriate (no error in fact was generated), leading to disruption of an otherwise intact action plan. Later evidence cast doubt on this view by showing that disruption from delayed auditory feedback still occurs when feedback content differs from what the participant planned to produce (Howell et al., 1983; Howell and Archer, 1984). However, the influential previous studies of Howell focused specifically on AAF that leads to asynchronies between perception and action which, as mentioned earlier, may involve a different neural network than that which is involved in processing altered pitch events. In the present study we addressed the link between AAF involving pitch content and error processing in a new way: By assessing whether auditory feedback influences the subjective experience of producing an error.

By demonstrating a biasing effect of AAF on error estimation, the present studies suggest that feedback may in fact be (mis)interpreted as an error signal. Thus, although feedback need not sound like an error to cause disruption, it may lead to misinterpretations of whether disruption has occurred. Such metacognitive aspects of performance may not be directly associated with one’s ability to maintain fluency. Recent research suggests that AAF may lead to a reduced experience of self-agency, along with increases in disruption, but that the two effects of AAF may operate in parallel (Couchman et al., 2012; cf. Logan and Crump, 2010). Along these lines it is important to note that increases in the disruptive effect of AAF were not associated with increases in the degree of overestimation. Furthermore, the biasing effect of AAF was subtle; if participants relied fully or even primarily on auditory feedback for error estimates the biasing effect would have been several orders of magnitude higher than was found. Thus, the present data suggest a modest role for auditory feedback in error estimates. That being said, the fact that any biasing effect at all was found is noteworthy, given that instructions to participants suggested that they ignore auditory feedback.

One aspect of the current methodology that is worth exploring further is the role of memory. For practical reasons, we asked participants to estimate error frequencies after a trial was finished, given that any attempt to estimate errors during a trial could introduce a new source of interference with performance. It is possible that the present effects reflect confusion of produced versus feedback-based errors in episodic memory. Specifically, participants may overestimate error frequencies after a trial because AAF interferes with one’s memory for the action plan.

### EFFECTS OF INTERMITTENT AAF

A methodological contribution of the present research is that it is the first study to our knowledge that employed AAF alterations with varying frequency on non-consecutive events. Past studies have included AAF manipulations on rare and temporally isolated events (e.g., Furuya and Soechting, 2010; Maidhof et al., 2010), which do not lead to significant increases in errors, or on consecutive events that span the entire sequence or a circumscribed subsequence (Pfordresher and Kulpa, 2011). The present study demonstrates that AAF disruption scales with the number of altered events and that these alterations need not be consecutive.

The fact that such intermittent alterations disrupt performance has important theoretical implications for the basis for AAF disruption. Pfordresher and Kulpa (2011) found that alterations to feedback content (pitch) only occurred after an extended sequence of AAF events (16 events in their study), in contrast to feedback asynchrony, which disrupted timing of production after only one AAF event. Based on those results, the authors speculated that effects of pitch alteration may occur because associations between planned events and sequence positions are gradually degraded after hearing several consecutive alterations to pitch. Although the present data do not refute this interpretation, they do suggest that the overall number of altered events may matter more than their temporal contiguity. Moreover, the present data are open to an alternate interpretation based on the idea that disruption occurs due to uncertainty concerning the outcomes of one’s actions (cf. Couchman et al., 2012).

### AAF AND VOCAL IMITATION

Along with original experiments on piano production, we also present a new correlational analysis of effects of AAF on singing, focusing on the role of individual differences in overall singing accuracy. The analysis we presented here, from data sets that were originally constructed to test different research questions, suggest that poor-pitch singers may in fact be less sensitive to AAF than are more accurate singers. This result suggests that poor-pitch singers may not use normal auditory feedback to guide production under normal circumstances, at least not in the same way as do more accurate singers. We propose that poor-pitch singers lack a properly functioning internal model of the auditory–vocal system and as such the predictions made by the forward model may be vague and/or distorted relative to actual auditory feedback. If so, then normal auditory feedback may for these participants have effects similar to altered feedback for more accurate singers, and the introduction of AAF may thus not change things much.

Other effects of feedback suggest that some poor-pitch singers may have difficulty using corrective feedback to guide production. Pfordresher and Brown (2007) found that poor pitch singers who mistuned by more than 100 cents on average actually performed worse when singing along with a recording of the target melody than when they sang alone. This result converges with the present data in suggesting that poor singers do not have a forward model that appropriately predicts the outcomes of their actions. Specifically, poor singers may not be able to correct their production based on differences between their own feedback and that of the target because their forward model cannot properly relate motor plans to the sound of their own voice.

## CONCLUSION

The experiments reported here demonstrated that participants use auditory feedback as a source of information about the accuracy of sequence production, though probably not the primary source of information. The use of auditory feedback for this purpose does not appear to rely on executive control but may instead reflect the more basic functioning of internal models for action planning. A secondary contribution of this research is that it shows disruptive effects of AAF do not require that manipulations be present consistently but instead scale with the relative frequency of altered events, although the use of feedback for error estimation was independent of AAF frequency. Future research will further evaluate the role of memory in the biasing effect of AAF on error estimation.

## Conflict of Interest Statement

The authors declare that the research was conducted in the absence of any commercial or financial relationships that could be construed as a potential conflict of interest.

## Footnotes

1 Follow-up analyses on musical experience revealed no consistent effects related to training. Thus, in the interest of brevity, we present analyses that are averaged across all participants.

2 Follow-up analyses on the effects of repeated trials showed significant learning effects, with repetitions reducing error rates and estimates of errors across all feedback conditions. However, the relationship between produced and estimated errors (the centerpiece of the present paper) did not vary across repetitions.

3 In both experiments reported here, AAF significantly slowed production and increased timing variability for the condition in which all possible events were altered. This result differs from previous studies showing a null effect of serially shifted AAF on timing (e.g., Pfordresher, 2003, 2005). The difference in the present data likely reflects the fact that participants needed to count produced errors, and experienced interference from AAF in this process.

4 We measured timing, as in Experiment 1, though again these data were not our primary focus. Across trials and participants, the mean IOI was very close to what was found for Experiment 1 (M = 324), though timing variability was lower in Experiment 2 (M CV = 0.09).
